# A framework to guide the implementation of lean management in emergency department

**DOI:** 10.1108/JHOM-01-2021-0035

**Published:** 2021-09-22

**Authors:** Anna Tiso, Maria Crema, Chiara Verbano

**Affiliations:** Department of Management and Engineering, University of Padova , Padova, Italy; Azienda ULSS N 2 Marca Trevigiana , Treviso, Italy

**Keywords:** Emergency department, Lean management, Lean healthcare, Literature review, Implementation framework

## Abstract

**Purpose:**

The paper aims at enriching the knowledge of the application of lean management (LM) in emergency department (ED), structuring the methodology for implementing LM projects and summarizing the relevant dimensions of LM adoption in ED.

**Design/methodology/approach:**

In accordance with the Preferred Reporting Items for Systematic Reviews and Meta-Analyses (PRISMA) statement, a systematic literature review has been performed, extracting a database of 34 papers. To answer the research purpose, a descriptive and content analyses have been carried out.

**Findings:**

The descriptive analysis demonstrates that the dealt topic is worldwide emerging and multidisciplinary as it arouses interest by medical and engineering communities. Despite the heterogeneity in the adopted methodology, a framework can be grasped from the literature review. It points out the phases and activities, the tools and techniques and the enablers to be considered for guiding the developing of LM project in ED.

**Originality/value:**

This paper provides a comprehensive overview on how to adopt LM in ED, contributing to fill in the gap emerged in the literature. From a practical perspective, this paper provides healthcare managers with a synthesis of the best managerial practices and guidelines in developing a LM project in ED.

## 1. Introduction

During the last decades, healthcare has assumed a priority role in international policy, becoming a decisive welfare indicator and pointing out the relevancy of improving operational efficiency to stay ahead in public service ( Antony *et al.* , 2019 ; Carter *et al.* , 2012 ). With the urge of rising health competitiveness, several states committed a great amount of resources and investment to increase performance, deeply damaged by the recent phenomena of international crisis and pandemic emergency that developed countries are facing. Indeed, the socio-demographic changes and economic issues have led worldwide healthcare to a sustainability problem, where aging population requires more sophisticated and expensive treatments despite heavy reductions on resources and budget ( Mousavi Isfahani *et al.* , 2019 ). National Health Systems are committed to maintain a balanced budget, in the face of a growing amount of people needing care due to the increase in life expectancy and in citizens' consciousness about their health conditions ( McIntosh *et al.* , 2014 ). In this regard, delivering a high quality assistance to customers while contracting costs represents one of the major challenges ( OECD, 2019 ). The integration of these complex concerns caused the crisis of the international healthcare sector, making it necessary to focus on this field. In particular, emergency department (ED) represents one of the most critical wards, afflicted by inefficient performances and unsatisfied patients. Literature shows how overcrowding, low quality care, excessive waiting times and high costs are the most problematic aspects affecting ED performances ( Bucci *et al.* , 2016 ). The rising importance acquired by health services from the citizens' point of view highlighted the need to overcome the relevant issues afflicting healthcare, to systematically improve its performance, planning a deep managerial change, rather than a simple cost cutting. In order to provide flexible and reactive responses to the above-mentioned phenomena, a process reorganization has been activated in health context, finding in engineering techniques the solution to improve its managerial and organizational structure achieving the pursued objectives in terms of efficiency and quality. Among the methods employed, lean management (LM) represents the most expanding approach, adopted by the 86% of health facilities ( Filser *et al.* , 2017 ; Henrique and Godinho Filho, 2020 ), in particular in EDs, surgery and laboratory ( D'Andreamatteo *et al.* , 2015 ). Considering the unique role of the ED and its impact on hospital performance, the current study focuses on the adoption of LM in this specific ward. ED has been considered the safety net of the healthcare system, but the increasing problem of crowding has strained this safety net to the “breaking point”, affecting the quality and access to care ( Hoot and Aronsky, 2008 ). The ED ability to promptly respond to the characteristics of urgency and emergency is penalized by inadequate services and coordination. Moreover, ED inefficiencies could critically impact on interdependent upstream and downstream processes along the patient pathway throughout the entire hospital system, translating bottlenecks to other wards. ED complexity deals with the high variety of pathways and with the great variability in demand and capacity caused by its peculiarities of urgency and emergency. This sort of unpredictability and diversity in clinical records make ED different from other departments and services, also highlighting the importance of intervening in this specific field. Indeed, in a context where crowding results in long waiting times, high rates of ED abandonment, delays in acute treatments and increased length of hospital stays, LM is definitely a preferred approach to reduce wastes mostly related to waiting, transportation and motion ( Daultani *et al.* , 2015 ) and to enhance quality of care.

Given the recent expansion of research on the topic, in the literature, there are still gaps in the operative and practical adoption of LM in ED. The experience heterogeneity, due to the novelty of the theme in research, but also in practice, hinders the recognition of a standardized methodology for applying LM in this setting; instead, different adoptable approaches to this phenomenon emerge from the literature. The lack of uniformity complicates the understanding of which tools, methods and principles are suitable for different processes and issues ( Régis *et al.* , 2018 ). Moreover, there are still little insights on the possibility to make the implementation procedure systematic in order to reduce the risk of failure. Many literature reviews exposed the state of publication in the application of LM in ED, focusing on tools, methods and results, nevertheless highlighting a remarkable difficulty in analyzing and reporting the implementation phase. In particular, only Holden (2011) provided a comprehensive review on LM adoption in ED, briefly presenting project development, deepening the effect of improvements on patient care and on employee satisfaction, outlining the contingency factors affecting the success of lean thinking efforts; on the contrary, the other authors ( Bucci *et al.* , 2016 ; Deblois and Lepanto, 2016 ; Mousavi Isfahani *et al.* , 2019 ) focused mainly on interventions and results on performances, investigating effects, facilitators and barriers to the implementation of LM in emergency units. However, an up-to-date overall literature review is missing. Hence, the current study aims to systematically analyze literature for enriching the knowledge on the application of LM in ED, focusing on the implementation of this methodology. In order to achieve that research objective, the analysis takes into consideration only case studies, investigating for each examined LM project the motivations and the goals, the application methodology, comprehensive of tools and tasks, the results in performances and the enabler factors. Thus, this literature review purposes to summarize the relevant aspects of LM adoption in ED, giving notable attention to the developed methodology for implementing LM improvement projects and the specific steps that it is composed by. Furthermore, based on the abovementioned systematic literature reviews on LM in EDs, it emerges the lack of recent research on the state of the art of this topic, as the latest paper included in the database of those studies dates from 2015. Therefore, the research motivation is based on the need to enhance the knowledge of lean healthcare adoption, both from an academic point of view by updating the existing systematic literature reviews focused on ED and from a practical and operative perspective, investigating the existence of a standardized and structured methodology for designing LM projects in ED.

The remainder of the article is structured as follows: a section entitled “Lean Management application in ED” with a description of the main concepts and development of LM in ED; a section dedicated to the followed “Methodology”, with all the details about the review methodology; a section with the “Results”, in order to present the key findings of the literature analysis; the “Results Discussion” to highlight the relevance of the obtained results inside the research topic. The final section concludes the article deriving implications and pointing out the directions for future research.

## 2. Lean management application in ED

Health LM refers to the application of lean thinking principles in healthcare organizations ( Borges *et al.* , 2019 ) with the purpose of creating a philosophy of process continuous improvement by either increasing customer value or reducing non-value adding activities, process variation and poor work conditions ( Radnor *et al.* , 2012 ). Lean healthcare has increasingly been adopted since 2000 ( Radnor *et al.* , 2012 ), demonstrating its potential in improving health performance in terms of productivity, flexibility, reactivity, efficiency, process capacity and quality, with positive effects on patient safety and mortality ( Abdallah and Alkhaldi, 2019 ; Crema and Verbano, 2015 ; Hallam and Contreras, 2018 ; Parkhi, 2019 ; Ramori *et al.* , 2021 ; Tlapa *et al.* , 2020 ). ED revealed to be the pioneer area in developing LM projects and the starting point of improvement processes ( Bucci *et al.* , 2016 ). Several authors report evidences of LM application in EDs, with improvement in patient flow and efficiency, reduction of waiting time and wastes and reinforcement of continuous improvement strategies as a key driver of the change process ( Al Owad *et al.* , 2018 ; Eller, 2009 ; Hitti *et al.* , 2017 ; Improta *et al.* , 2018 ; White *et al.* , 2014 ). Moreover, successful results have been documented in decreased length of stay and turnaround time, in addition to enhanced quality and satisfaction ( Allaudeen *et al.* , 2017 ; Arbune *et al.* , 2017 ; El Sayed *et al.* , 2015 ; Sánchez *et al.* , 2018 ; Vashi *et al.* , 2019 ).

LM application in healthcare and ED is still in its early stages compared to its development in manufacturing sector, due to the complexity of the health system and the initial skepticism toward the adoption of an industrial methodology ( Radnor *et al.* , 2012 ). Lean healthcare implementation must take into consideration the intrinsic differences between manufacturing and health industries, as in healthcare the value is provided to customers through a service. This aspect influences patient satisfaction and mutually gets influenced by human behaviors. Consequently, processes reveal to be partially unpredictable, depending on personal reality perception, decision-making approach and the established relation with the environment, constantly affecting the output delivering. Additionally, the complexity of healthcare systems constitutes a deterrent in demonstrating successful improvements evidence, due to deeply embedded cultural norms and organizational customs ( Antony *et al.* , 2019 ; Currie *et al.* , 2007 ). However, given the urgency of improving ED critical performances to promptly respond to the emerging needs, there is a great interest in understanding how to properly apply LM in ED, generating guidelines and identifying the enablers’ factors for a successful implementation. In this regard, assuming that LM is a philosophy rather than an ordinary method, a cultural system change and specific facilitator conditions are necessary for integrating and incorporating lean principles into the long-term strategy ( Schonberger, 2018 ). Besides, LM adoption demonstrated to be strictly related to the application context, underlining the requirement of adapting techniques and procedures to the specific condition and needs.

## 3. Methodology

In order to analyze how LM projects have been developed in ED, overcoming the abovementioned shortcomings, a systematic literature review has been conducted.

**Figure 1:**
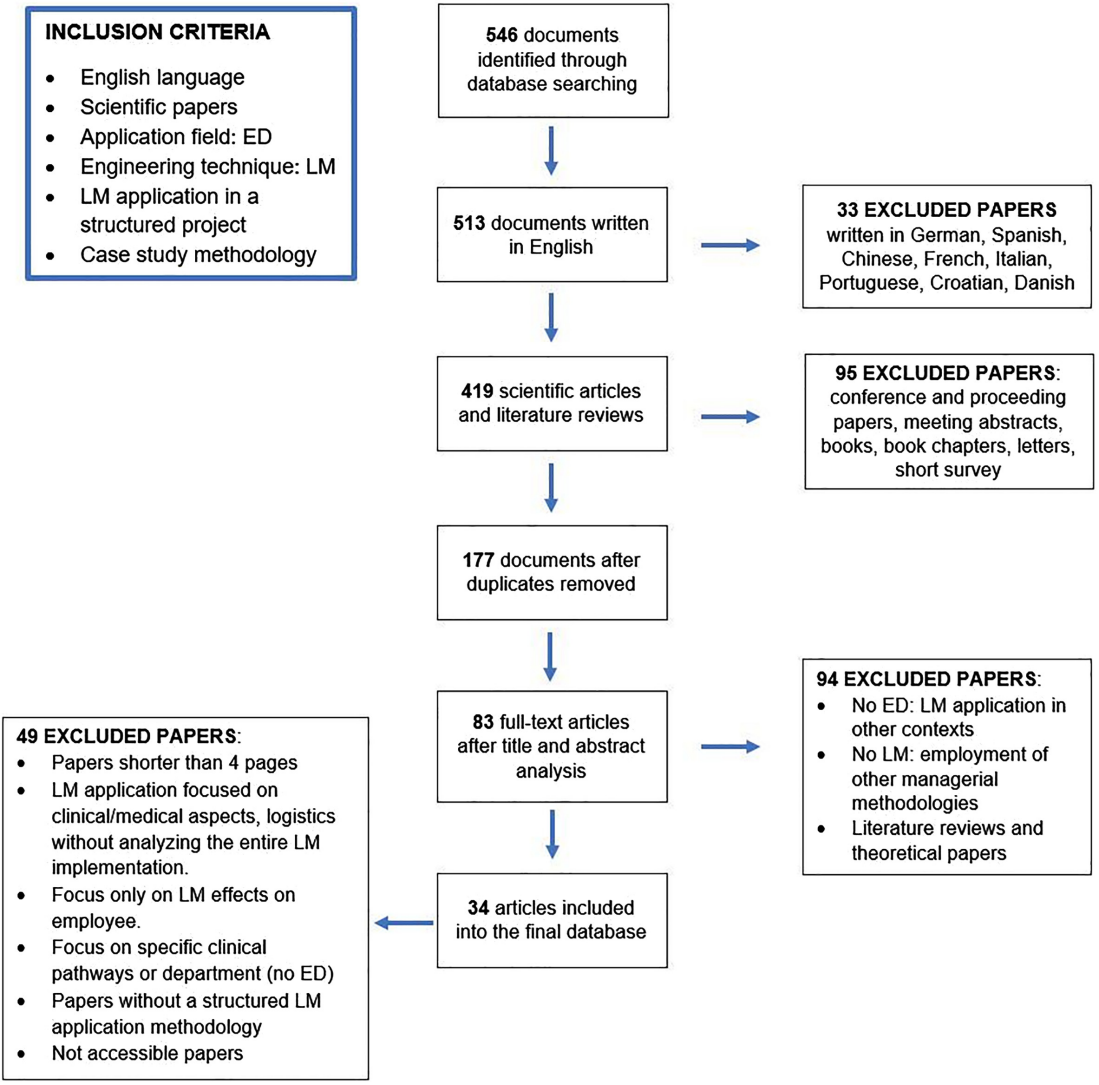
Selection process and inclusion criteria

The current literature review (finished in January 2021) has been accomplished in accordance with the Preferred Reporting Items for Systematic reviews and Meta-Analyses (PRISMA) statement, which consists of a 27-item checklist and a flow diagram that guided this study in searching and selecting the most appropriate publications. The study was developed in 6 consecutive phases and followed 6 inclusion criteria ( Figure 1 ) with the aim of accurately collecting and analyzing all the empirical evidence answering the research question. The first step concerned with the exploration of three academical databases, Scopus, PubMed and Web of Science, through a keyword searching, in order to identify published articles regarding LM applications in EDs. Each scientific database has been consulted using the following 9 different combinations of keywords: “lean management,” “lean thinking,” “lean healthcare,” “lean production,” “kaizen,” “patient flow,” “lean methodology,” “lean tools,” “lean techniques,” associated with the Boolean operator AND to “(emergency department OR emergency room)”. In particular, the research referred to “Topic” field in Web of Science, to “All the fields” in PubMed, while it has been limited to “Article Title, Abstract, Keywords” in Scopus. A total amount of 546 papers has been identified and screened according to the predetermined inclusion and exclusion criteria, derived from the specific research objectives. At the beginning, the inclusion criteria allowed to focus on articles written in English, excluding 33 papers in other languages, and on scientific articles, deleting 95 conference papers, books or book chapters and short surveys. After removing 242 duplications, 177 articles were submitted to a title and abstract analysis, in order to preliminarily evaluate their consistency to the investigation question and to the inclusion criteria, eliminating 78 documents not referring to ED as the application context or to LM as the main managerial methodology adopted. Moreover, only case studies have been included in the database, excluding 16 literature reviews, considered not relevant to address the research goal of extrapolating practices and tools from empirical successful LM application in ED. Finally, 83 full-text articles accessed for eligibility have been reviewed, adopting the exclusion criteria to narrow them down. First of all, the adequacy of the project setting has been considered, excluding papers focused on specific clinical pathways, that only partially impacted on ED performance. The objective of these papers consisted in improving a particular dimension of performance in order to achieve better performance locally, inside the specific pathway or department (e.g. laboratory turnaround times, radiology transport cycle times). However, the effect of the interventions on ED performance was out of their scope, keeping the analysis very narrowed. These cases demonstrated to be not relevant for the final database, given the research interest in studying the LM applications in ED and their impact on the performance of this department. Secondly, the adoption of a structured approach in applying LM has been evaluated, excluding papers without a robust project organization and development, an in-depth description of the followed tasks and a proper and recognizable tools application. Lastly, LM projects whose objectives resulted to be not coherent to the research goal have been removed: papers focused on detailed clinic or medical aspects, on logistics or only on employee satisfaction and perception of the improvement process, without analyzing the entire LM implementation, have been considered not adequate to grasp useful information to define a LM framework in ED.

Thanks to this structured selection process, presented in Figure 1 , 34 articles have been retained for the final database that has been studied performing a descriptive and a content analysis.

The descriptive analysis provided a qualitative evaluation of the extracted database, highlighting the publication year trend, the most committed countries to the topic, the subject area distribution of each journal and the quality assessment of the journals.

The content analysis was carried out for each paper considering, according to the research goal, its motivations and objectives, the applied methodology (comprehensive of the followed tasks and the employed tools), the achieved results and finally the key success factors.

## 4. Results

### 4.1 Descriptive analysis of the literature

The final database consists of 34 scientific articles, developed following the case study methodology and concerned with the application of LM tools and techniques in ED. With the purpose of providing an overview of the literature on the research topic, a descriptive analysis has been performed on the selected database. Most articles were written by more than 4 authors, but only 10 authors collaborated in writing more than one paper, highlighting the innovation of the topic and the margin for further study, besides the lack of a scientific and consolidated community of experts specialized on the theme. Furthermore, the novelty of the topic is confirmed by the publication trend, increasing from 2014 until 2020, period in which 71% of papers in the database have been published, while no articles have been found before 2008. The subject area has been analyzed according to Scopus criteria, revealing that 79% of the journals belonged to the branch of medicine and 18% to “industrial and manufacturing engineering.” Those results proved the relevance of the selected papers in answering the research question, since they denoted the application of managerial techniques to the health system and emphasized the interest of both scientific communities in the future development of the topic.

This distribution has been endorsed by the analysis of the 29 journals in the database, where “BMC Health Services Research” and “Journal for Healthcare Quality” were the most frequent, with 3 papers each. The quality of the database has been assessed through the bibliometric indicators, ascertaining the prestige of the journals thanks to the Scimago Journal Rank (SJR), with 75% of the journal papers published in Q1 and Q2 journals, and to the Web of Science Impact Factor, higher than 1.200 for the 62% of the journals.

The performed descriptive analysis revealed the geographical distribution of LM projects described in the selected papers. The LM adoption in 17 different countries confirmed its worldwide diffusion, both in industrialized and in developing countries, demonstrating how the application of this managerial methodology operates also in low-resource health systems. USA, with 12 papers (35% of the total database), is a promoter of LM implementation, followed by Italy (12%), while minor efforts have been observed in UK, Lebanon and Saudi Arabia.

As the setting is concerned, the analyzed projects were implemented only in ED, except for 3 papers, focused on other departments, such as radiology or laboratory, strictly connected to ED and mutually influencing their performance.

### 4.2 Content analysis of the literature

With the aim of assessing LM implementation in EDs, the content analysis has been performed inspecting: the motivations underlying its adoption, the pursued objectives, the followed methodology, the adopted LM tools, the achieved results and the key success factors.

#### 4.2.1 LM projects motivations and objectives

**Figure 2:**
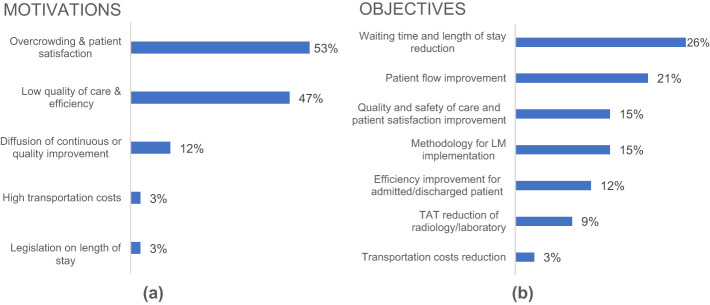
Motivations (2a) and objectives (2b) of the analyzed LM projects

The study of the project motivations demonstrated how the crisis of the healthcare sector played a primary role in pulling EDs towards an engineering reorganization process ( Figure 2 ). In this regard, several authors justified the LM project with the need of managing overcrowding and improving patient satisfaction through shorter waiting times, while other scholars motivated the LM project with the necessity of delivering higher quality of care and efficiency reducing costs. The 12% of the authors, by applying LM in ED, strived for building and spreading throughout the organization a new strategy of continuous and systematic improvement.

The LM methodology has been applied with the aim to improve and manage patient flow, identifying root causes and wastes ( Al Owad *et al.* , 2018 ; Alowad *et al.* , 2021 ; Ben-Tovim *et al.* , 2008 ; Carney *et al.* , 2020 ; Improta *et al.* , 2018 ; White *et al.* , 2014 ), but also to enhance the quality and the safety of care, assuring transparency and reliability to achieve an adequate level of patient and employee satisfaction ( Cookson *et al.* , 2011 ; Dickson *et al.* , 2009a, b ; Kane *et al.* , 2015 ; Matt *et al.* , 2018 ; Rees, 2014 ), albeit containing costs. Several papers focused on reduction of waiting time and delays, in order to increase efficiency and limit overcrowding, evaluating how LM principles and tools impacted on specific variables, such as length of stay (LOS) in ED ( Alexander *et al.* , 2020 ; Arbune *et al.* , 2017 ; Bal *et al.* , 2017 ; Chan *et al.* , 2014 ; Elamir, 2018 ; Eller, 2009 ; Furterer, 2018 ; Nicholas, 2012 ; Sánchez *et al.* , 2018 ; Vashi *et al.* , 2019 ), radiology or laboratory turnaround time (TAT) ( Hitti *et al.* , 2017 ; Sanders and Karr, 2015 ; Verbano and Crema, 2019 ), duration and costs of patient transportation in ED ( Chiarini, 2013 ). According to other authors, the crisis of ED was due to factors external to ED control, such as bed availability in other wards and ambulance services that negatively affect ED internal performance. For this reason, Ng *et al.* (2010) concentrated only on discharged patients with the objective of enhancing ED efficiency and productivity; on the contrary, Allaudeen *et al.* (2017) and Wolak *et al.* (2020) aimed at reducing the LOS of patient admission, considered the most critical phase in both clinical and organizational perspective. Finally, in other instances, the case study has been conducted with the pursue of explaining and divulging a clear and structured methodology for standardizing the application of LM in ED (still missing in the current literature), promoting an holistic view in applying LM ( Naik *et al.* , 2012 ), highlighting the key aspect of implementation by comparing different case studies ( Rees, 2014 ), evaluating its development in a low resource context ( Carter *et al.* , 2012 ) and testing a framework that integrates LM and resilience engineering ( Rosso and Saurin, 2018 ).

#### 4.2.2 The methodology employed in the LM projects

Once examined the motivations and the objectives of each LM project, the employed methodology has been analyzed, investigating similarities and differences among the case studies. First of all, it is remarkable that LM represented the only managerial approach adopted, except Bal *et al.* (2017) , White *et al.* (2014) , who combined LM with queuing theory, demand-capacity matching and simulation models, and Rosso and Saurin (2018) , jointly using resilience engineering and LM. Second, in every paper of the database, it is possible to pinpoint a LM methodology developed step-by-step in a chronological sequence of the followed activities and of the employed tools and techniques. Although the acknowledged relevancy of adopting a structured method in improvement projects, the several different approaches emerging from the analyzed literature underlined how the value of identifying a standardized and unique practice is still underestimated. A significant heterogeneity has been observed in the methodology adopted in the 34 case studies, depending on the specific pursued objectives, resource availability and the degree of awareness of LM. However, the analysis of the LM application phases showed the presence of common features in carrying out the different projects, as it is represented in Table 1 .

The initial phase coincided with the preliminary activities of planning and defining the project at a high level. All the analyzed papers, except White *et al.* (2014) , reported a detailed description of the accomplished tasks that encompassed the identification of the project scope, the definition of specific goals and of the adequate strategy, often after a preliminary analysis of the critical areas at a global level. Indeed, Sanders and Karr (2015) and Arbune *et al.* (2017) pointed out the importance of defining precisely the focus of the project before its execution, setting measurable and time-related objectives in order to guarantee data integrity and allow improvement monitoring.

The creation of multidisciplinary teams represented another fundamental activity in this phase, since in all projects the composition and the selection of the work group have been deepened. The project team varied according to the specific needs and to the employee proneness to change, integrating together workers with different functional expertise, ranging from clinicians and nurses to technicians and clerks, engineers and hospital managers. Moreover, in seven studies ( Arbune *et al.* , 2017 ; Carney *et al.* , 2020 ; Dickson *et al.* , 2009a ; Improta *et al.* , 2018 ; Naik *et al.* , 2012 ; Ng *et al.* , 2010 ; Vashi *et al.* , 2019 ) an external quality improvement facilitator or lean consultant flanked the project team by guiding and training on LM principles and practices. In other cases, teams have been supported by hospital personnel, expert in LM and employed in organizational units dedicated to process improvement and organizational management.

In the analyzed literature, after this first step, quantitative and qualitative data have been collected (baseline performance of the process, input and output metrics, location of causes of the problem, rate of occurrence of issues and cycle time data) to map the critical pathways identified during the preliminary analysis. Indeed, according to LM methodology, mapping the processes allows to quantify value adding and non-value adding activities, cycle times, delays and employed resources. The authors presented a wide variety of techniques to harvest data on pathways: numerical data mining of ED activities, timing and patient accesses; interviews and questionnaires to assess the satisfaction of patients and employees and to grasp insights from the frontline staff's experience; Gemba waste walks that are direct observations on the field with the aim of studying the ED dynamics, measuring the actual activities duration, detecting wastes. The combination of data analysis and process mapping aimed to identify the critical areas to be improved and to quantify their inefficiencies. Frequently in the studied LM projects, the mapping phase led to a further waste analysis, focusing on non-value adding activities and bottlenecks, with the purpose of identifying the root causes of the criticalities ( *muda* and root cause analysis). Table 2 shows the *mudas* identified by the authors of the database.

The prioritization of the problems dealt mainly with their impact on efficiency and safety. In some cases, instead, the mapping phase resulted insufficient to the purpose of identifying the source of wastes, requiring additional data gathering, including direct observations, surveys and mathematical and statistical analysis.

Only in 5 studies the future state mapping has been performed and among them, Bal *et al.* (2017) with the aim to prevent errors during the implementation phase and to achieve the consensus among the team on the planned actions.

In several studies the next step in developing LM project was the generation of improvement ideas based on the results achieved in the previous analysis. The main technique adopted in this phase is the brainstorming, involving the whole team in actively participating by sharing and discussing proposals for improvement. As it is underlined by some authors ( Table 1 ), the priority and the feasibility of the suggested solutions should be evaluated in order to focus efforts on the most relevant ones in relation to the prioritization of wastes.

In many cases, the improvement interventions consisted of small and simple actions that implied few resources and risks and were within reach of the frontline staff without the need of external assistance. Sometimes the above-mentioned solutions have been applied through Kaizen events, including pilot tests, sometimes instead Rapid Process Improvement Workshops (RPIW) has been employed to speed and lean the improvement process. Sporadic cases did not report the actual application of the interventions, but only discussed the future implementation planning ( Al Owad *et al.* , 2018 ; Alowad *et al.* , 2021 ; Bal *et al.* , 2017 ; Carter *et al.* , 2012 ; Cookson *et al.* , 2011 ; Matt *et al.* , 2018 ; Ng *et al.* , 2010 ; Nicholas, 2012 ).

The last steps dealt mainly with maintaining the gains achieved with the project, through standards creation and maintenance. According to Eller (2009) , the process standardization allowed to enhance employee engagement and improve the quality of care for ED patients. Several approaches have been adopted to introduce and control work standardization, consisting of in continuously monitoring key performance indicators (KPIs) and sharing real-time data on project progress with the team and the frontline staff involved.

#### 4.2.3 Tools and techniques adopted in the LM projects

Throughout the project development, various LM tools and techniques have been employed according to the specific needs and objectives, but also depending on the phase and activity of the project development; they are gathered in Table 3 .

**Figure 3:**
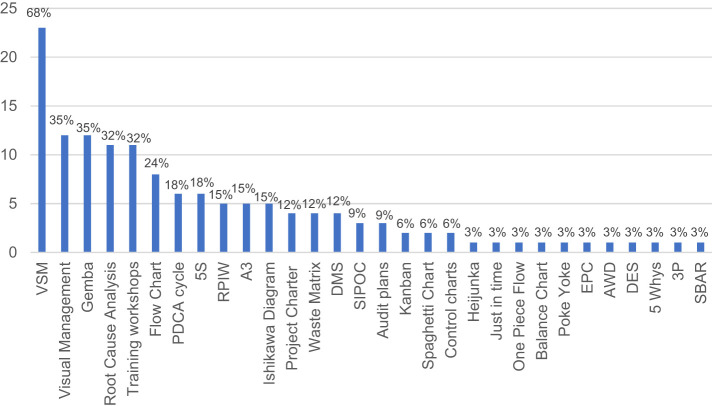
Employed LM tools and techniques

As reported in Figure 3 , the most employed technique was the VSM, used mainly in the current state mapping phase, followed by visual management techniques, Gemba waste walks, training workshops during kaizen events and root cause analysis and. Moreover, the PDCA cycle resulted to be successfully executed in the 18% of cases as an iterative control tool, while among the implementation techniques, 5S appeared to be the most utilized.

#### 4.2.4 Results obtained by the LM projects

**Figure 4:**
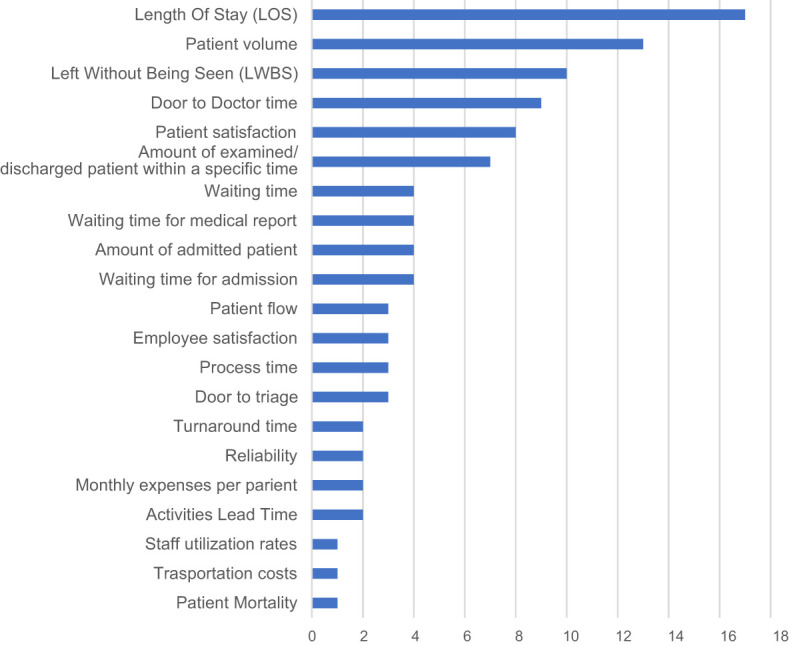
Obtained results

The results achieved by each LM project have been studied during the content analysis with the purpose of evaluating the success of the improvement process and of the methodology applied. First and foremost, all the papers registered the achievements through KPI monitoring sessions, except for 7 articles ( Al Owad *et al.* , 2018 ; Alowad *et al.* , 2021 ; Carter *et al.* , 2012 ; Cookson *et al.* , 2011 ; Nicholas, 2012 ; Rosso and Saurin, 2018 ; Wolak *et al.* , 2020 ). Even if the current analysis confirmed the benefits accomplished by the LM adoption, data gathering and monitoring were inaccurate in several case studies, limiting the possibility to generalize LM success. Indeed, some studies neglected before and after comparison of KPIs, other studies collected not detailed or incomplete data on the obtained outcomes; finally, just sporadic cases applied statistical tests to control results and to correlate the obtained results with the applied interventions ( Alexander *et al.* , 2020 ; Carney *et al.* , 2020 ; Mazzocato *et al.* , 2012 ; Sánchez *et al.* , 2018 ; Sanders and Karr, 2015 ; Vashi *et al.* , 2019 ). Only one paper ( Chan *et al.* , 2014 ) reported a worsening in LOS due to an increased rate of admitted patients that negatively impacted on ED waiting times. The variables most frequently improved in LM projects are reported in Figure 4 . In particular, the majority of cases achieved LOS reduction, in light of patient volume growth and of the LWBS (left without being seen) decrease, demonstrating a superior ability in managing the demand-capacity matching, improving therefore ED performance at a global level. Indeed, lower LOS translates into minor times (door-to-triage time, door-to-doctor time, reporting time, admission waiting time) and into increased efficiency in carrying out visits and value adding activities.

#### 4.2.5 Key factors in the LM projects developed in ED

Finally, the reviewed literature allowed to identify the key factors enabling a successful implementation of LM in EDs, summarized in Table 4 . Based on the emerging importance of orienting the system toward a continuous improvement strategy, many authors considered the inclination to change of the organization a key factor to build a flexible healthcare system, inspired by the progress and ready to modify embedded habits and behaviors. In order to fulfill the purpose of building a shared and accepted organizational culture based on LM, several authors highlighted the relevance of four factors in developing a LM project in ED: the creation of multidisciplinary project teams, trained on LM principles and skilled in problem-solving; the empowerment of the ED frontline staff in an active participation to the improvement process, improving staff relations; the involvement in the work group of the stakeholders of the project, included patient through the voice of customer, to enable consensual decision-making; the essential support of leadership. The role of leadership sustained the creation and diffusion of LM philosophy among employees, acting as a role model in the paradigm shift towards a bottom-up perspective of continuous improvement long-term view. This approach encouraged the integration of ED doctors and nurses inside the project team with the aim of entrusting them with individual responsibility in achieving the pursued project objectives and reinforcing their commitment in collaborating in the improvement process, reducing the internal opposition toward change. Moreover, the employees' involvement in the project team generated a remarkable contribution in assuring, thanks to their experience on the field, the feasibility and the real practicability of the planned interventions, organized in small and simple changes, effectively developable by ED frontline staff. This structuring of improvement ideas represented a key success factor for many authors, allowing to reduce the resources needed, to celebrate the achievements and to mitigate a radical change, by introducing small improvements that are long-term sustainable ( Improta *et al.* , 2018 ).

Other authors highlighted the importance of a structured implementation methodology for the success of LM improvement projects, suitable for the specific context and adapted to the local needs. Among them, some cases promoted the adoption of a holistic approach to consider the effects related to the improvement project on the entire value stream and to act synergically both on the upstream and downstream process, breaking down departmental barriers. Moreover, Rosso and Saurin (2018) highlighted the relevance of considering also patient safety, besides efficiency, among healthcare performance improvement, integrating resilience engineering with LM methods.

Finally, according to plenty of authors, the continuous monitoring activities constituted the key factor for the successful maintenance of improvement, promoting the usefulness of meetings, feedback, standard work, visual management and the PDCA cycle to share the good practices developed and emphasize the progress, generating consensus towards the project.

## 5. Results discussion

The empirical evidence provided by the current study highlighted how the recent LM adoption in healthcare settings is still in its early stage, confirming the novelty of the phenomenon. The findings of this review suggested a wide approach heterogeneity and a lack of a standardized methodology to develop a project to apply LM in ED. Indeed, the differences in structuring the LM projects in each case study is remarkable, as well as the absence of implementation guidelines. However, many studies developed a similar path, composed by a sequence of tasks that resulted to be frequently conducted in the analyzed literature.

First of all, the initial phase dealt with objective and resource planning, team creation (and sometimes training) and the preliminary current state analysis. These activities allowed to define the boundaries of the project, defining the specific goals and their feasibility in relations to time and resource constraints, and contributed to understand the focus of the improvement process at a high level. Second, the project continued with the precise and detailed identification of critical processes, by analyzing information regarding timings, access, performances and using these data to map the current state of the pathways selected for the LM intervention. Based on the data extracted by the mapping phase, the project team dedicated itself to waste analysis, detecting the non-value adding activities and correlating them to *mudas* , and to the subsequent root cause analysis. The primary focus of lean philosophy is to eliminate *mudas* with the final goal of boosting health performance. Most of lean implementations detected waiting time as the major waste in ED, also becoming the main target to reduce in order to improve the quality of care and productivity. As highlighted by the analysis of *mudas* , also inventory waste, overproduction and defects and disservice frequently affect ED processes. In addition, the lack of standard procedures guiding the ED processes causes increased waiting times, errors and repetitions of unnecessary work. For this reason, since several inefficiencies are caused by a combination of wastes rather than a single *muda* , a deeper analysis is needed to inspect the root cause of inefficiencies. Consequently, some improvement actions were generated in order to eliminate the source of waste and, once verified their practicability, implemented in the field. Finally, the last phase regarded the standard work creation and maintenance, by continuously monitoring the targets and by developing protocols and tools to facilitate the team in sustaining the change.

This methodology has been adopted in different contexts and pursuing diverse project goals; in the totality of the considered cases, it led to the achievement of successful results in terms of performance improvement. Therefore, it can be concluded that the above-mentioned procedure resulted effective for developing LM projects in ED. However, increased performance does not necessarily entail LM success, considering also that only in a few studies statistical assessments of results have been executed to reduce the risk of endogeneity. Indeed, the introduction of control variables would assure that high performance is achieved thanks to LM implementation, eliminating the contribution of other factors. Moreover, it is widely acknowledged that LM implementation methodology *per se* does not guarantee the success of an improvement project, as LM is considered a philosophy made up by a bundle of hard and soft practices. This means that an adequate LM application methodology is necessary but insufficient to determine the accomplishment of the project: it should be integrated by an appropriate employment of hard tools (e.g. VSM, Flow chart, Kanban) coherently accompanied by soft practices (e.g. continuous improvement, leadership support, employee involvement) and guided by a LM organizational culture. Among the analyzed case studies, different hard tools, techniques and practices have been applied; in particular VSM, Kaizen event, root cause analysis and visual management techniques, but also many soft practices, such as the involvement of workers and stakeholders into the project team, the support of leadership, the development of employee training programs and finally the attempts of orientating the system toward a continuous improvement. However, given the scarcity of detailed descriptions on techniques application process, its results are difficult to affirm whether the LM tools have been adapted correctly to the health context, or have been employed in the correct phase of project development.

With regard to the soft practices adopted, the analyzed literature emphasized their contribution inside the improvement project to the point that they have been frequently considered the key factors enabling the success of a LM project in ED. Moreover, with an opportune combination of key factors, like engaged frontline workers, long-term leadership commitment and workforce flexible to change, LM could represent the methodology to continuously improve patient flow, service and performance in the ED ( Dickson *et al.* , 2009a , b ).

Hence, the evidence provided by the studied literature gathers that the real strength of LM lies in its approach to problem-solving and its underlying philosophy, based on worker involvement, on a supportive leadership oriented to quality, on creation of solid processes and on introduction of small cycles of changes.

**Figure 5:**
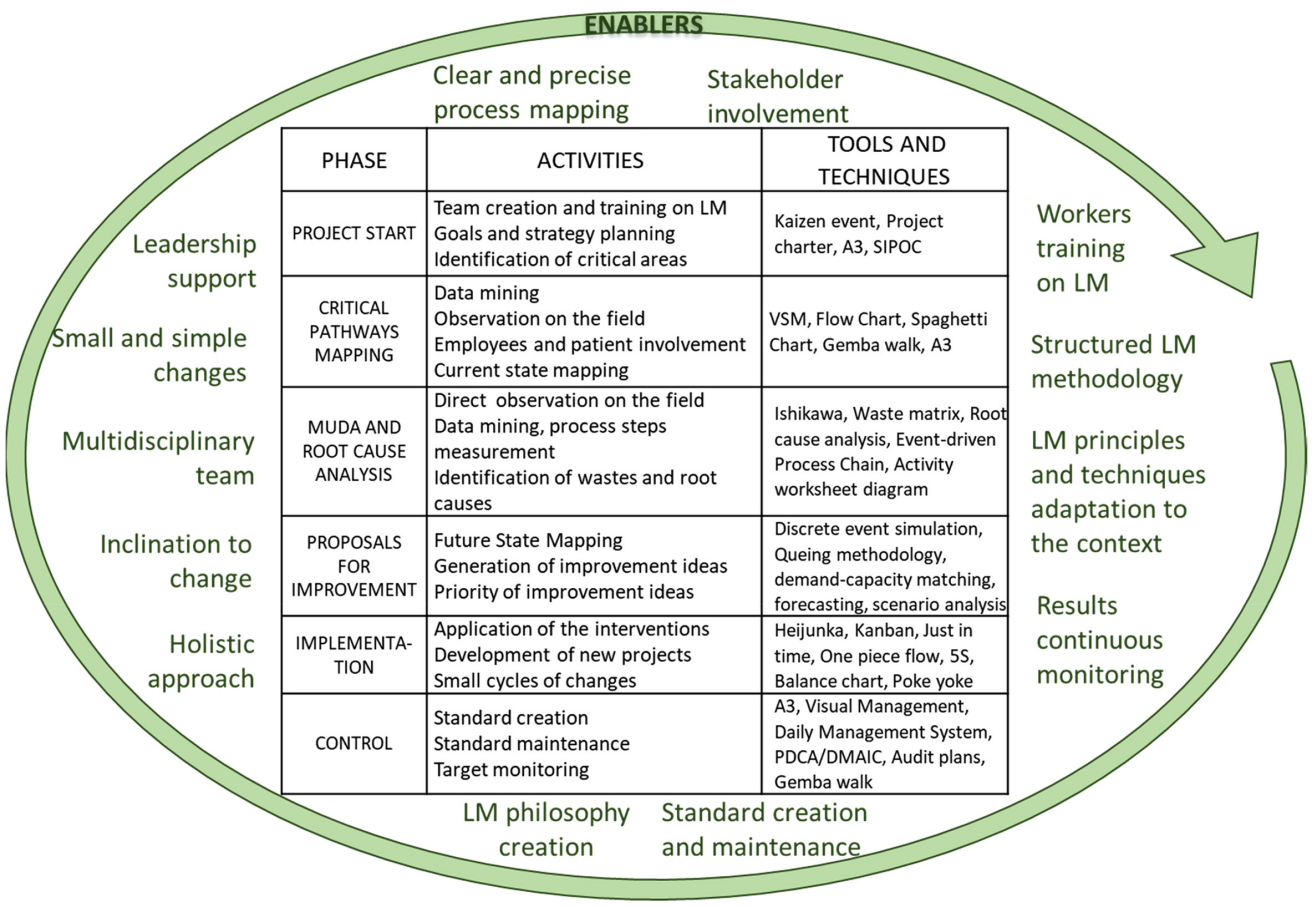
The emerging framework for the application of LM in ED

The following Figure 5 reports the resulting framework that illustrates phases, activities, tools and techniques and the enabling factors for the implementation of LM in ED.

## 6. Conclusions

LM application in healthcare is increasingly demonstrating its capability in facing ED overcrowding and patient dissatisfaction by creating a patient-oriented system that enhances efficiency, quality and safety of care. The current study outlined the most relevant aspects of the adoption of LM in ED, aiming to provide a contribution from an academic point of view, by presenting a comprehensive overview, missing in the current literature, on how LM projects have been structured in ED in the last years. The final purpose consists in seeking the existence of a standardized framework for implementation in healthcare, and eventually, with further studies, exploring its applicability in diverse settings, conducting a comparison between ED results and other wards.

From a managerial perspective, the analysis of the published case studies allowed to identify the best managerial practices and guidelines to support healthcare managers in developing successful LM projects. The latter should be performed, coherently with LM philosophy, by adopting an improvement approach founded on employee involvement, on creation of solid processes and on introduction of small cycles of changes.

In conclusion, the current literature review highlighted how a shared and common procedure in executing LM projects still lacks, but at the same time, the diversity of experiences provided evidence on the different approaches and techniques adoptable to implement LM in EDs. Based on this finding, further research is needed to apply and confirm the grasped framework for the development of LM projects in ED. Single and multiple case studies should be conducted following the suggested framework, highlighting adaptations necessary to implement LM in diverse contexts. These future studies would help to identify the different methodologies based on the peculiarities of diverse contexts, contributing to fill in the gap underlined by scholars (e.g. Régis *et al.* , 2018 ; Borges *et al.* , 2019 ; Henrique and Godinho Filho, 2020 ; Ramori *et al.* , 2021 ; Parkhi, 2019 ): the lack of understanding of which methodologies in terms of activities, procedures, tools and principles are suitable for different processes and issues. Indeed, the limited number of papers extracted and the heterogeneity of the detected aspects in each case study represents a limitation for this literature review. Therefore, the managerial and academic state of the art should be kept updated with future studies. In addition, the lack of statistical assessment of LM project results increased the risk of misunderstanding the effects of each tool and technique in healthcare facilities. For this reason, future research could be performed to analyze the statistical correlation between LM and the achievements in performance improvement. The introduction of control variables in that research would assure that high performance is achieved thanks to LM implementation, eliminating the contribution of other factors. In addition, other research could be performed to deeply analyze the correlation between the identified enabling factors and the objectives, activities, tools and techniques of LM projects. This research would contribute to address the request of understanding which behaviors and soft skills impact on the success or failure of a LM project ( Antony *et al.* , 2019 ). Studies which adopt simulation techniques could be helpful to test in advanced the potentialities of LM in the abovementioned future research.

Little attention has been focused on the creation and spreading of the LM philosophy throughout the system, ignoring the importance of sharing an organizational culture to strengthen the adhesion towards the change. In this respect, further research should be conducted with the purpose of analyzing which aspects of the organizational culture result to be essential for LM successful implementation in healthcare. Moreover, other research is needed to investigate how to effectively introduce the new system culture oriented toward kaizen into health organizations.

In the future, it could be valuable to conduct other research to define how the sustainability of a LM project could be guaranteed over time.

Furthermore, future studies that compare the LM application in ED with its adoption in other wards in the same hospital could be interesting in order to recognize similarities and differences in such implementations.

At the end, as highlighted also by Rosso and Saurin (2018) and Crema and Verbano (2016) , few research are still performed on LM and patient safety, but it could be useful to take advantage of integrating LM with other approaches, such as for instance clinical risk management.

Therefore, besides identifying gaps in the literature and providing a framework to develop LM projects in ED, this paper encourages future research in order to confirm the obtained results and to strength the adoption of new organizational and managerial approaches in ED, with the final aim of improving the healthcare management and the satisfaction of internal personnel and of the community in general.

## Figures and Tables

**Table 1:** Phases and activities of project development

Phase	Activities	Case studies
Project Start	Team creation and training on LM principles	( Al Owad *et al.* , 2018 ; Alowad *et al.* , 2021 ; Allaudeen *et al.* , 2017 ; Arbune *et al.* , 2017 ; Bal *et al.* , 2017 ; Ben-Tovim *et al.* , 2008 ; Carney *et al.* , 2020 ; Carter *et al.* , 2012 ; Chan *et al.* , 2014 ; Chiarini, 2013 ; Cookson *et al.* , 2011 ; Dickson *et al.* , 2009a , b ; El Sayed *et al.* , 2015 ; Elamir, 2018 ; Eller, 2009 ; Furterer, 2018 ; Hitti *et al.* , 2017 ; Improta *et al.* , 2018 ; Kane *et al.* , 2015 ; Matt *et al.* , 2018 ; Mazzocato *et al.* , 2012 ; Naik *et al.* , 2012 ; Ng *et al.* , 2010 ; Nicholas, 2012 ; Rosso and Saurin, 2018 ; Sánchez *et al.* , 2018 ; Sanders and Karr, 2015 ; Vashi *et al.* , 2019 ; Verbano and Crema, 2019 ; Wolak *et al.* , 2020 )
	Goals and strategy planning	( Alexander *et al.* , 2020 ; Arbune *et al.* , 2017 ; Carney *et al.* , 2020 ; Carter *et al.* , 2012 ; Furterer, 2018 ; Kane *et al.* , 2015 ; Matt *et al.* , 2018 ; Mazzocato *et al.* , 2012 ; Ng *et al.* , 2010 ; Sanders and Karr, 2015 ; Vashi *et al.* , 2019 ; Verbano and Crema, 2019 ; Wolak *et al.* , 2020 )
	Preliminary analysis of the current state: Identification of critical areas of the hospital through brainstorming	( Alexander *et al.* , 2020 ; Arbune *et al.* , 2017 ; Bal *et al.* , 2017 ; Ben-Tovim *et al.* , 2008 ; Carter *et al.* , 2012 ; Chan *et al.* , 2014 ; Chiarini, 2013 ; Dickson *et al.* , 2009a , b ; El Sayed *et al.* , 2015 ; Furterer, 2018 ; Hitti *et al.* , 2017 ; Improta *et al.* , 2018 ; Kane *et al.* , 2015 ; Matt *et al.* , 2018 ; Naik *et al.* , 2012 ; Ng *et al.* , 2010 ; Nicholas, 2012 ; Rosso and Saurin, 2018 ; Sánchez *et al.* , 2018 ; Sanders and Karr, 2015 ; Vashi *et al.* , 2019 ; Wolak *et al.* , 2020 )
Critical pathways mapping	Data mining	( Alowad *et al.* , 2021 ; Allaudeen *et al.* , 2017 ; Arbune *et al.* , 2017 ; Bal *et al.* , 2017 ; Carter *et al.* , 2012 ; El Sayed *et al.* , 2015 ; Eller, 2009 ; Hitti *et al.* , 2017 ; Improta *et al.* , 2018 ; Matt *et al.* , 2018 ; Ng *et al.* , 2010 ; Sanders and Karr, 2015 ; Vashi *et al.* , 2019 ; Verbano and Crema, 2019 )
	Observation on the field	( Al Owad *et al.* , 2018 ; Alowad *et al.* , 2021 ; Allaudeen *et al.* , 2017 ; Chiarini, 2013 ; Cookson *et al.* , 2011 ; Dickson *et al.* , 2009a ; Rosso and Saurin, 2018 ; Sanders and Karr, 2015 ; Vashi *et al.* , 2019 ; Verbano and Crema, 2019 )
	Employees and patients' involvement (surveys/brainstorming/interviews)	( Al Owad *et al.* , 2018 ; Alowad *et al.* , 2021 ; Alexander *et al.* , 2020 ; Chiarini, 2013 ; Elamir, 2018 ; Furterer, 2018 ; Improta *et al.* , 2018 ; Matt *et al.* , 2018 ; Ng *et al.* , 2010 ; Rosso and Saurin, 2018 ; Verbano and Crema, 2019 )
	Current state mapping	( Al Owad *et al.* , 2018 ; Alowad *et al.* , 2021 ; Alexander *et al.* , 2020 ; Allaudeen *et al.* , 2017 ; Arbune *et al.* , 2017 ; Bal *et al.* , 2017 ; Ben-Tovim *et al.* , 2008 ; Carney *et al.* , 2020 ; Carter *et al.* , 2012 ; Chan *et al.* , 2014 ; Chiarini, 2013 ; Cookson *et al.* , 2011 ; Dickson *et al.* , 2009a , b ; El Sayed *et al.* , 2015 ; Elamir, 2018 ; Eller, 2009 ; Furterer, 2018 ; Hitti *et al.* , 2017 ; Improta *et al.* , 2018 ; Kane *et al.* , 2015 ; Matt *et al.* , 2018 ; Mazzocato *et al.* , 2012 ; Naik *et al.* , 2012 ; Ng *et al.* , 2010 ; Nicholas, 2012 ; Rosso and Saurin, 2018 ; Sánchez *et al.* , 2018 ; Sanders and Karr, 2015 ; Vashi *et al.* , 2019 ; Verbano and Crema, 2019 ; White *et al.* , 2014 ; Wolak *et al.* , 2020 )
Muda and root cause analysis	Observation on the field	( Arbune *et al.* , 2017 ; Carter *et al.* , 2012 ; Elamir, 2018 ; Furterer, 2018 ; Improta *et al.* , 2018 ; Nicholas, 2012 )
	Data collection (patient survey)	Alowad *et al.* (2021)
	Data mining, process steps measurement	( Bal *et al.* , 2017 ; Carter *et al.* , 2012 ; Dickson *et al.* , 2009a , b ; Elamir, 2018 ; Furterer, 2018 ; Improta *et al.* , 2018 ; Matt *et al.* , 2018 ; Mazzocato *et al.* , 2012 ; Ng *et al.* , 2010 ; Nicholas, 2012 ; Sánchez *et al.* , 2018 ; Sanders and Karr, 2015 ; White *et al.* , 2014 ; Wolak *et al.* , 2020 )
	Identification of wastes and root causes	( Al Owad *et al.* , 2018 ; Alowad *et al.* , 2021 ; Alexander *et al.* , 2020 ; Allaudeen *et al.* , 2017 ; Arbune *et al.* , 2017 ; Bal *et al.* , 2017 ; Carney *et al.* , 2020 ; Carter *et al.* , 2012 ; Chiarini, 2013 ; Cookson *et al.* , 2011 ; Dickson *et al.* , 2009a , b ; El Sayed *et al.* , 2015 ; Elamir, 2018 ; Eller, 2009 ; Furterer, 2018 ; Hitti *et al.* , 2017 ; Improta *et al.* , 2018 ; Kane *et al.* , 2015 ; Matt *et al.* , 2018 ; Mazzocato *et al.* , 2012 ; Ng *et al.* , 2010 ; Nicholas, 2012 ; Rosso and Saurin, 2018 ; Sánchez *et al.* , 2018 ; Sanders and Karr, 2015 ; Vashi *et al.* , 2019 ; Verbano and Crema, 2019 ; White *et al.* , 2014 ; Wolak *et al.* , 2020 )
	Prioritization of wastes/problems	( Alexander *et al.* , 2020 ; Cookson *et al.* , 2011 ; Elamir, 2018 ; Kane *et al.* , 2015 ; Matt *et al.* , 2018 ; Ng *et al.* , 2010 ; Rosso and Saurin, 2018 ; White *et al.* , 2014 )
Proposals for improvement	Future state mapping	( Alowad *et al.* , 2021 ; Bal *et al.* , 2017 ; Matt *et al.* , 2018 ; Naik *et al.* , 2012 ; Ng *et al.* , 2010 ; Sanders and Karr, 2015 )
	Generation of improvement ideas	( Alowad *et al.* , 2021 ; Alexander *et al.* , 2020 ; Allaudeen *et al.* , 2017 ; Arbune *et al.* , 2017 ; Bal *et al.* , 2017 ; Ben-Tovim *et al.* , 2008 ; Carney *et al.* , 2020 ; Carter *et al.* , 2012 ; Chan *et al.* , 2014 ; Chiarini, 2013 ; Cookson *et al.* , 2011 ; Dickson *et al.* , 2009a , b ; Elamir, 2018 ; Eller, 2009 ; Furterer, 2018 ; Hitti *et al.* , 2017 ; Improta *et al.* , 2018 ; Kane *et al.* , 2015 ; Matt *et al.* , 2018 ; Mazzocato *et al.* , 2012 ; Naik *et al.* , 2012 ; Ng *et al.* , 2010 ; Nicholas, 2012 ; Rosso and Saurin, 2018 ; Sánchez *et al.* , 2018 ; Sanders and Karr, 2015 ; Vashi *et al.* , 2019 ; Verbano and Crema, 2019 ; White *et al.* , 2014 ; Wolak *et al.* , 2020 )
Implementation	Application of the interventions	( Alexander *et al.* , 2020 ; Carney *et al.* , 2020 ; Chan *et al.* , 2014 ; Chiarini, 2013 ; Dickson *et al.* , 2009a , b ; El Sayed *et al.* , 2015 ; Elamir, 2018 ; Eller, 2009 ; Hitti *et al.* , 2017 ; Improta *et al.* , 2018 ; Rosso and Saurin, 2018 ; Sánchez *et al.* , 2018 ; Sanders and Karr, 2015 ; Verbano and Crema, 2019 ; Wolak *et al.* , 2020 )
	Development of new projects	( Allaudeen *et al.* , 2017 ; Kane *et al.* , 2015 ; Naik *et al.* , 2012 ; Sanders and Karr, 2015 ; Vashi *et al.* , 2019 )
	Small cycles of changes	( Arbune *et al.* , 2017 ; Ben-Tovim *et al.* , 2008 ; Mazzocato *et al.* , 2012 )
Control	Interventions pilot test	Carney *et al.* (2020)
	Standard creation	( Allaudeen *et al.* , 2017 ; Ben-Tovim *et al.* , 2008 ; Dickson *et al.* , 2009a ; Eller, 2009 ; Furterer, 2018 ; Improta *et al.* , 2018 ; Kane *et al.* , 2015 ; Mazzocato *et al.* , 2012 ; Naik *et al.* , 2012 ; Ng *et al.* , 2010 ; Sánchez *et al.* , 2018 ; Sanders and Karr, 2015 ; Vashi *et al.* , 2019 ; Wolak *et al.* , 2020 )
	Standard maintenance	( Allaudeen *et al.* , 2017 ; Ben-Tovim *et al.* , 2008 ; Kane *et al.* , 2015 ; Sánchez *et al.* , 2018 ; Sanders and Karr, 2015 ; Vashi *et al.* , 2019 )
	Target monitoring	( Alexander *et al.* , 2020 ; Allaudeen *et al.* , 2017 ; Arbune *et al.* , 2017 ; Dickson *et al.* , 2009a , b ; El Sayed *et al.* , 2015 ; Eller, 2009 ; Furterer, 2018 ; Hitti *et al.* , 2017 ; Improta *et al.* , 2018 ; Naik *et al.* , 2012 ; Ng *et al.* , 2010 ; Rosso and Saurin, 2018 ; Sánchez *et al.* , 2018 ; Sanders and Karr, 2015 ; Verbano and Crema, 2019 ; Wolak *et al.* , 2020 )

**Table 2:** Muda identified during the waste analysis

Muda	Description	Examples	References
Waiting time	Wait for request and technician arrival to accept the patient, due to lack of communication	The information system does not warn the radiology technician that the ED has sent a radiological examination request, nor that the patient has arrived	( Allaudeen *et al.* , 2017 ; Elamir, 2018 ; Verbano and Crema, 2019 )
	Delays for referral	-The patient stay was prolonged by delays in referral from ED and psychiatry staff -Staff waiting for results	( Alexander *et al.* , 2020 ; Cookson *et al.* , 2011 ; Elamir, 2018 ; Verbano and Crema, 2019 )
	Delays at triage	During peak hours, volume too great for one triage nurse to handle	Vashi *et al.* (2019)
	Wait for physician/nurse	Patients waiting for assessment	( Chiarini, 2013 ; Cookson *et al.* , 2011 ; Mazzocato *et al.* , 2012 ; Sánchez *et al.* , 2018 ; Verbano and Crema, 2019 )
	Wait for inpatient beds	ED patient waiting for inpatient bed availability	( Carter *et al.* , 2012 ; Elamir, 2018 ; Vashi *et al.* , 2019 )
	Delays	-Delayed handover of updates -Delays caused by handoffs	( Alexander *et al.* , 2020 ; Allaudeen *et al.* , 2017 )
Transport	Inadequate patient transportation	Patients moved from one box to another depending on staff preferences	( Sánchez *et al.* , 2018 ; Verbano and Crema, 2019 )
	Long transportation	Long distances between services	Cookson *et al.* (2011)
	Unnecessary patient transportation	Moving ED patients to separate areas for admit holding	Carter *et al.* (2012)
Inventory	Referrals	Following the logic first in first out for reporting of radiological examinations causes queues in ED	Verbano and Crema (2019)
	Excessive/poor inventory	-Excessive stock supply to ensure availability -Unavailable stock or out of useable date	( Carter *et al.* , 2012 ; Cookson *et al.* , 2011 )
	Underutilized employee	No engagement in process redesign	Carter *et al.* (2012)
	Useless documentation	Multiple unnecessary patient forms	( Carter *et al.* , 2012 ; Vashi *et al.* , 2019 )
	Unnecessary material	Disarray in nurses' charts	Sánchez *et al.* (2018)
	Batching tests	Ordering tests for more than one patient at once	Sánchez *et al.* (2018)
	Batching patient	-Queue at triage, radiology -Staff placing and preparing more than one patient at once	( Chiarini, 2013 ; Sánchez *et al.* , 2018 ; Vashi *et al.* , 2019 ; Verbano and Crema, 2019 )
Motions	Doctor/nurse movements	-Doctor seeking nurse (or vice versa), or patients -Staff walking back and forward for the photocopier	( Cookson *et al.* , 2011 ; Sánchez *et al.* , 2018 ; Vashi *et al.* , 2019 )
	Patient movements	Following triage, veterans returned to waiting room even if open bed available	Vashi *et al.* (2019)
	Movements of administrative personnel	Lengthy distance between administrative process steps	Carter *et al.* (2012)
Over-Production	Unnecessary first visit	In some cases, the first visit consists only of a radiological examination request, and it is therefore useless for the patient to wait for it	Verbano and Crema (2019)
	Over-triaging	Unnecessary triage phase	Vashi *et al.* (2019)
	Unnecessary activity	Radiology acceptance	Verbano and Crema (2019)
	Unnecessary tests	Ordering unnecessary investigations	Cookson *et al.* (2011)
	Duplication of information	Recording the same information multiple times	Cookson *et al.* (2011)
Errors or disservices	Disservice in transportation	Many patients arrive in wrong departments or are forced to repeatedly ask for information, due to a lack of indications	Verbano and Crema (2019)
	Defects	Incorrect surgical procedure, medication error	Carter *et al.* (2012)
	Bed issues	No empty beds, bed occupied when not needed	Vashi *et al.* (2019)
	Inadequate treatment	Antibiotics for viral infection	Carter *et al.* (2012)
	Lack of communication	Difficulties in communicating updates	( Alexander *et al.* , 2020 ; Vashi *et al.* , 2019 ; Verbano and Crema, 2019 )
Processing	Role confusion	No clear definition of roles and responsibilities	Alexander *et al.* (2020)
	No alternate processes during peak	Volume too great for available capacity	Vashi *et al.* (2019)
	Lack of coordination	Overlapping assessments	Alexander *et al.* (2020)
	Reworks	-Doctor/nurse ordering tests or medications in a fragmented manner -Reassessment of patient by several members of the staff	( Allaudeen *et al.* , 2017 ; Cookson *et al.* , 2011 ; Sánchez *et al.* , 2018 )
	Lack of protocols	-No standards for using hallways, for patient assignments (doctors' self-assignment of patients) -Lack of standard procedures for handoffs	( Allaudeen *et al.* , 2017 ; Vashi *et al.* , 2019 )

**Table 3:** LM tools and techniques

Activity	Tool and technique	References
Team Training	Workshops/meetings	( Alowad *et al.* , 2021 ; Allaudeen *et al.* , 2017 ; Ben-Tovim *et al.* , 2008 ; Carter *et al.* , 2012 ; Dickson *et al.* , 2009a , b ; Matt *et al.* , 2018 ; Naik *et al.* , 2012 ; Ng *et al.* , 2010 ; Rosso and Saurin, 2018 ; Sánchez *et al.* , 2018 ; Sanders and Karr, 2015 )
Planning	Project charter	( Furterer, 2018 ; Matt *et al.* , 2018 ; Sanders and Karr, 2015 ; Vashi *et al.* , 2019 )
	A3	Arbune *et al.* (2017)
	Suppliers inputs process outputs customers (SIPOC)	( Carter *et al.* , 2012 ; Furterer, 2018 ; Sanders and Karr, 2015 )
MAPPING	Value stream map (VSM)	( Alowad *et al.* , 2021 ; Arbune *et al.* , 2017 ; Bal *et al.* , 2017 ; Carney *et al.* , 2020 ; Carter *et al.* , 2012 ; Chan *et al.* , 2014 ; Chiarini, 2013 ; Cookson *et al.* , 2011 ; Dickson *et al.* , 2009a , b ; El Sayed *et al.* , 2015 ; Eller, 2009 ; Hitti *et al.* , 2017 ; Improta *et al.* , 2018 ; Kane *et al.* , 2015 ; Matt *et al.* , 2018 ; Naik *et al.* , 2012 ; Ng *et al.* , 2010 ; Nicholas, 2012 ; Rosso and Saurin, 2018 ; Sánchez *et al.* , 2018 ; Vashi *et al.* , 2019 ; Verbano and Crema, 2019 ; White *et al.* , 2014 )
	Flow chart	( Al Owad *et al.* , 2018 ; Alowad *et al.* , 2021 ; Chan *et al.* , 2014 ; Dickson *et al.* , 2009a ; Elamir, 2018 ; Eller, 2009 ; Sánchez *et al.* , 2018 ; Sanders and Karr, 2015 )
	Spaghetti chart	( Chiarini, 2013 ; Nicholas, 2012 )
Data Gathering	Gemba waste walk	( Al Owad *et al.* , 2018 ; Alowad *et al.* , 2021 ; Arbune *et al.* , 2017 ; Carter *et al.* , 2012 ; Chiarini, 2013 ; El Sayed *et al.* , 2015 ; Elamir, 2018 ; Improta *et al.* , 2018 ; Nicholas, 2012 ; Rosso and Saurin, 2018 ; Sanders and Karr, 2015 )
	A3	( Al Owad *et al.* , 2018 ; Alowad *et al.* , 2021 )
Waste and cause identification	Ishikawa	( Al Owad *et al.* , 2018 ; Alowad *et al.* , 2021 ; Carter *et al.* , 2012 ; El Sayed *et al.* , 2015 ; Furterer, 2018 )
	5-Whys	Wolak *et al.* (2020)
	Waste matrix	( Carter *et al.* , 2012 ; Sánchez *et al.* , 2018 ; Sanders and Karr, 2015 ; Verbano and Crema, 2019 )
	Root cause analysis	( Allaudeen *et al.* , 2017 ; Cookson *et al.* , 2011 ; El Sayed *et al.* , 2015 ; Elamir, 2018 ; Eller, 2009 ; Furterer, 2018 ; Improta *et al.* , 2018 ; Mazzocato *et al.* , 2012 ; Sanders and Karr, 2015 ; Vashi *et al.* , 2019 ; Verbano and Crema, 2019 )
	Event-driven process chain (EPC)	Al Owad *et al.* (2018)
	Activity worksheet diagram (AWD)	Chiarini (2013)
Proposals for improvement	3P (production preparation process)	Nicholas (2012)
	SBAR (situation, background, assessment, recommendation) communication tool	Wolak *et al.* (2020)
	Discrete event simulation (DES)	Bal *et al.* (2017)
	Queuing methodology, demand-capacity matching, forecasting, scenario analysis	White *et al.* (2014)
Implementation	RPIW	( Allaudeen *et al.* , 2017 ; Kane *et al.* , 2015 ; Naik *et al.* , 2012 ; Sanders and Karr, 2015 ; Vashi *et al.* , 2019 )
	Heijunka	Hitti *et al.* (2017)
	Kanban	( Hitti *et al.* , 2017 ; Ng *et al.* , 2010 )
	Just in time	Ng *et al.* (2010)
	One piece flow	Sánchez *et al.* (2018)
	5S	( Bal *et al.* , 2017 ; Ben-Tovim *et al.* , 2008 ; Improta *et al.* , 2018 ; Kane *et al.* , 2015 ; Sánchez *et al.* , 2018 ; White *et al.* , 2014 )
	Balance chart	Improta *et al.* (2018)
	Poke yoke	Sanders and Karr (2015)
Control	A3	( Bal *et al.* , 2017 ; Carter *et al.* , 2012 )
	Visual management	( Allaudeen *et al.* , 2017 ; Arbune *et al.* , 2017 ; Bal *et al.* , 2017 ; Carter *et al.* , 2012 ; Hitti *et al.* , 2017 ; Improta *et al.* , 2018 ; Kane *et al.* , 2015 ; Matt *et al.* , 2018 ; Ng *et al.* , 2010 ; Sánchez *et al.* , 2018 ; Sanders and Karr, 2015 ; Vashi *et al.* , 2019 )
	Daily management system	( Allaudeen *et al.* , 2017 ; Arbune *et al.* , 2017 ; Kane *et al.* , 2015 ; Vashi *et al.* , 2019 )
	Plan do check Act (PDCA)/Plan do study Act (PDSA) as control cycle	( Allaudeen *et al.* , 2017 ; Furterer, 2018 ; Mazzocato *et al.* , 2012 ; Ng *et al.* , 2010 ; Sánchez *et al.* , 2018 ; Vashi *et al.* , 2019 )
	Control charts	( Carney *et al.* , 2020 ; Sanders and Karr, 2015 )
	Audit plans	( Ng *et al.* , 2010 ; Vashi *et al.* , 2019 ; Verbano and Crema, 2019 )
	Gemba waste walk	Kane *et al.* (2015)

**Table 4:** Enabler factors for LM projects

Enabler factors	References
Multidisciplinary team	( Carter *et al.* , 2012 ; Cookson *et al.* , 2011 ; Dickson *et al.* , 2009a , b ; Furterer, 2018 ; Kane *et al.* , 2015 ; Mazzocato *et al.* , 2012 ; Ng *et al.* , 2010 ; Nicholas, 2012 ; Rees, 2014 ; Sánchez *et al.* , 2018 ; Verbano and Crema, 2019 )
Leadership support	( Ben-Tovim *et al.* , 2008 ; Carter *et al.* , 2012 ; Cookson *et al.* , 2011 ; El Sayed *et al.* , 2015 ; Furterer, 2018 ; Kane *et al.* , 2015 ; Naik *et al.* , 2012 ; Ng *et al.* , 2010 ; Rees, 2014 ; Verbano and Crema, 2019 )
Stakeholder involvement in the project team	( Al Owad *et al.* , 2018 ; Alowad *et al.* , 2021 ; Alexander *et al.* , 2020 ; Allaudeen *et al.* , 2017 ; Arbune *et al.* , 2017 ; Cookson *et al.* , 2011 ; El Sayed *et al.* , 2015 ; Furterer, 2018 ; Improta *et al.* , 2018 ; Naik *et al.* , 2012 ; Nicholas, 2012 ; Rees, 2014 ; Verbano and Crema, 2019 )
Small and simple changes	( Alexander *et al.* , 2020 ; Arbune *et al.* , 2017 ; Carter *et al.* , 2012 ; Dickson *et al.* , 2009a , b ; El Sayed *et al.* , 2015 ; Improta *et al.* , 2018 ; Naik *et al.* , 2012 ; Ng *et al.* , 2010 ; Vashi *et al.* , 2019 )
Inclination to change	( Ben-Tovim *et al.* , 2008 ; El Sayed *et al.* , 2015 ; Furterer, 2018 ; Improta *et al.* , 2018 ; Rees, 2014 )
LM principles and techniques adaptation to the context	( Carter *et al.* , 2012 ; Dickson *et al.* , 2009a , b ; El Sayed *et al.* , 2015 ; Sánchez *et al.* , 2018 ; Vashi *et al.* , 2019 )
Continuous monitoring of results	( El Sayed *et al.* , 2015 ; Furterer, 2018 ; Verbano and Crema, 2019 )
Structured methodology in developing the project	( Allaudeen *et al.* , 2017 ; Improta *et al.* , 2018 ; Naik *et al.* , 2012 ; Rees, 2014 )
Focus on flow	( Dickson *et al.* , 2009a ; Ng *et al.* , 2010 ; Vashi *et al.* , 2019 )
Workers training on LM	( Ng *et al.* , 2010 ; Verbano and Crema, 2019 )
Effective communication	( Arbune *et al.* , 2017 ; Naik *et al.* , 2012 ; Wolak *et al.* , 2020 )
Holistic approach	( Matt *et al.* , 2018 ; Ng *et al.* , 2010 )
Standard creation and maintenance	( Ben-Tovim *et al.* , 2008 ; Mazzocato *et al.* , 2012 ; Wolak *et al.* , 2020 )
Creation and spreading of LM philosophy and adoption of a continuous improvement approach	( Al Owad *et al.* , 2018 ; Alowad *et al.* , 2021 ; Cookson *et al.* , 2011 ; Rees, 2014 )
Clear and precise process mapping	( Alowad *et al.* , 2021 ; Improta *et al.* , 2018 )
